# Role of Intestinal Bacteria in Gliadin-Induced Changes in Intestinal Mucosa: Study in Germ-Free Rats

**DOI:** 10.1371/journal.pone.0016169

**Published:** 2011-01-13

**Authors:** Jana Cinova, Giada De Palma, Renata Stepankova, Olga Kofronova, Miloslav Kverka, Yolanda Sanz, Ludmila Tuckova

**Affiliations:** 1 Department of Immunology, Institute of Microbiology v.v.i., Academy of Sciences of the Czech Republic, Prague, Czech Republic; 2 Microbial Ecophysiology and Nutrition Group, Institute of Agrochemistry and Food Technology (IATA), National Spanish Research Council (CSIC), Valencia, Spain; CNRS - Université Aix-Marseille, Institut de Biologie du Développement de Marseille Luminy, France

## Abstract

**Background and Aims:**

Celiac disease (CD) is a chronic inflammatory disorder of the small intestine that is induced by dietary wheat gluten proteins (gliadins) in genetically predisposed individuals. The overgrowth of potentially pathogenic bacteria and infections has been suggested to contribute to CD pathogenesis. We aimed to study the effects of gliadin and various intestinal bacterial strains on mucosal barrier integrity, gliadin translocation, and cytokine production.

**Methodology/Principal Findings:**

Changes in gut mucosa were assessed in the intestinal loops of inbred Wistar-AVN rats that were reared under germ-free conditions in the presence of various intestinal bacteria (enterobacteria and bifidobacteria isolated from CD patients and healthy children, respectively) and CD-triggering agents (gliadin and IFN-γ) by histology, scanning electron microscopy, immunofluorescence, and a rat cytokine antibody array. Adhesion of the bacterial strains to the IEC-6 rat cell line was evaluated *in vitro*.

Gliadin fragments alone or together with the proinflammatory cytokine interferon (IFN)-γ significantly decreased the number of goblet cells in the small intestine; this effect was more pronounced in the presence of *Escherichia coli* CBL2 and *Shigella* CBD8. *Shigella* CBD8 and IFN-γ induced the highest mucin secretion and greatest impairment in tight junctions and, consequently, translocation of gliadin fragments into the lamina propria. *Shigella* CBD8 and *E. coli* CBL2 strongly adhered to IEC-6 epithelial cells. The number of goblet cells in small intestine increased by the simultaneous incubation of *Bifidobacterium bifidum* IATA-ES2 with gliadin, IFN-γ and enterobacteria. *B. bifidum* IATA-ES2 also enhanced the production of chemotactic factors and inhibitors of metalloproteinases, which can contribute to gut mucosal protection.

**Conclusions:**

Our results suggest that the composition of the intestinal microbiota affects the permeability of the intestinal mucosa and, consequently, could be involved in the early stages of CD pathogenesis.

## Introduction

Mucosal surfaces of the gastrointestinal tract are continuously exposed to environmental stimuli. The intestinal epithelium constitutes the largest and most important barrier against external environmental agents and has two critical functions: to prevent the entry of harmful intraluminal microorganisms, antigens, and toxins and to enable the selective translocation of dietary nutrients and electrolytes into circulation.

One of the basic properties of gut-associated lymphoid tissue (GALT) is oral tolerance (unresponsiveness) to harmless components of microbiota and diet. Inappropriate immunological reactions against food proteins, such as wheat components, can lead to the breakdown of oral tolerance and the development of intestinal immune disorders.

Celiac disease (CD) is a chronic immune-mediated enteropathy of small intestine that is triggered by dietary wheat gluten, or related rye and barley proteins in genetically susceptible individuals. More than 90% of patients carry HLA-DQ2/8 antigens. The expression of these high-risk haplotypes in general population, however, is 20% to 30%, only 3% to 5% of whom develop CD. The involvement of genes for cytokines interleukin (IL)-21 and IL-2 in CD pathogenesis has been reported recently [1]–[5]. The ingestion of gluten is the key environmental cause linked to the symptoms of CD, but also infections and the composition of the intestinal microbiota might play a role in CD pathogenesis [6]–[10]. Gluten proteins are partially hydrolyzed by peptidases in the gastrointestinal tract, so the gluten (gliadin)-derived peptides can cross the epithelium and be converted by tissue transglutaminase (TG) 2 into negatively charged peptides that have higher affinity for HLA-DQ2 and HLA-DQ8 molecules. Gliadin peptides are presented by dendritic cells (DC) to CD4+ α/β T lymphocytes in the jejunum. Activated gliadin-specific T cells up-regulate type 1 and 2 cytokines that activate other cell types. The substantial increase in interferon (IFN)-γ promotes a proinflammatory environment and the activation of tissue enzymes, including metalloproteinases and TG2, which are involved in CD pathogenesis [11]–[16].

The outermost barrier of gut mucosa is formed by a single layer of epithelial cells covered by thick, viscous and relatively impermeable gel layer produced by goblet cells – mucus. This mucus layer prevents direct contact between enteric pathogens and epithelial cell surfaces, contains binding sites for resident microbiota and maintains high concentrations of secretory IgA to prevent pathogens from attaching and entering. Moreover, Paneth cells producing various antimicrobial peptides or lysozymes strengthen the first-line of defense against harmful agents [17]–[19].

The integrity and function of the intestinal epithelium depend on a protein network that joins epithelial cells and consists of transmembrane complexes: tight junctions (TJs), adherens junctions, and desmosomes. TJs are present in most apical regions, where they selectively regulate the paracellular passage of ions and solutes and prevent the translocation of luminal antigens, microorganisms, and their toxins. TJs are formed by integral membrane proteins, primarily occludins and claudins. Claudins, a family of at least 24 proteins, are expressed in specific tissues; claudins 1-5 are expressed in the gut intestine. Occludins and claudins contain a binding domain for a complex of proteins - the zonula occludens (ZO-1, ZO-2, and ZO-3) - which is linked to the actin cytoskeleton and signaling proteins. Increased permeability of the epithelial barrier has been proposed to increase one's predisposition to intestinal inflammation and gastrointestinal diseases, including CD. Gluten and its component, gliadin were shown to alter the expression of TJ proteins and TJ-associated ZO-1 and stimulate the production of zonulin [20]–[23].

Recently, the potential role of the microbiota in CD pathogenesis has attracted attention. Indigenous commensal microbiota is involved in the resistance to infection not only through their direct interaction with pathogenic bacteria but also through their influence on the host immune system. The microbiota of CD patients showed different composition in feces and duodenal biopsy specimens compared with healthy controls, characterized by a preferential increase in the proportions of *Bacteroides* and *E. coli* with virulence genes and by a reduction in *Bifidobacterium* proportions [6]–[10], [24]–[26].

In this study, we examined the effect of gliadin and the proinflammatory cytokine IFN-γ on the intestinal barrier in rat intestinal loops in the presence of potentially pathogenic enteric bacteria isolated from CD patients or a *Bifidobacterium s*train isolated from healthy controls. The effects of these stimuli on mucosal barrier (TJs), its architecture, the number of goblet cells, adhesion, gliadin translocation, and cytokine secretion were compared.

## Materials and Methods

### Ethics Statement

All animal experiments were approved by the Laboratory Animal Care and Use Committee of the Institute of Microbiology v.v.i., Academy of Sciences of the Czech Republic, approval ID: 244/2009.

### Gliadin fragments

Peptic fragments of gliadin (Sigma, St Louis, MO) were prepared on a pepsin agarose gel (ICN, Biomedicals, Ohio) as described [27], [28]. Protein concentrations were measured by bicinchoninic acid assay (BCA Protein assay, Pierce, Rockford, IL). All reagents were tested by E-toxate test for lipopolysaccharide (LPS) (Sigma, St. Louis, MO) and were below the limit of detection (2 pg/ml).

### Cell culture and stimulation

The rat normal small intestine epithelial cell line (IEC)-6 was purchased from American Type Culture Collection (ATCC, CRL-1592, Manassas, VA) and was grown in 45% Dulbecco's Modified Eagle Medium (DMEM) Glutamax and 45% RPMI 1640, containing 10% inactivated (56°C for 30 min) fetal bovine serum (FBS) (Gibco, Barcelona, Spain), 100 µg/ml streptomycin, 100 U/ml penicillin, and 0.1 U/ml insulin (Sigma, St. Louis, MO) at 37°C and 5% CO_2_. Cells were stimulated with bacteria and gliadin fragments (100 µg).

### Bacterial strains and culture conditions

The following strains were used: *Bifidobacterium bifidum* IATA-ES2 (CECT 7365), *Shigella* CBD8, and *Escherichia coli* CBL2. *B. bifidum* IATA-ES2 was isolated from the feces of healthy babies and identified as described previously [29], [30]. *E. coli* CBL2 and *Shigella* CBD8 were isolated from celiac patients and identified as described by Sanchez et al. [26]. The *Shigella* strain was included to exemplify the possible effects of an actual intestinal pathogen in this disease context.

Bifidobacteria were grown routinely in de Man, Rogosa, and Sharpe (MRS) broth (Scharlau Chemie SA, Barcelona, Spain) with 0.05% (w/v) cysteine and incubated at 37°C under anaerobic conditions (AnaeroGen; Oxoid, Basingstoke, UK) for 22 h. Enterobacteria were grown routinely in Violet Red Bile Dextrose (VRBD) agar (Scharlau Chemie SA, Barcelona, Spain) at 37°C for 24 h under aerobic conditions.

Cells were harvested by centrifugation (6000× g for 15 min) at the stationary growth phase, washed 2 times with PBS, and resuspended in PBS that contained 20% glycerol. Aliquots of these suspensions were frozen in liquid nitrogen and stored at −80°C until use. The number of live cells after storage was determined as colony-forming units (CFUs) on MRS-C, Schadler, or VRBD agar after 48 h incubation under optimal conditions. For all strains, more than 90% cells were alive on thawing, and no significant differences were observed during storage (4 months). One fresh aliquot was thawed for each new experiment to avoid variabilities in live bacterial cell numbers between experiments.

### Bacterial adhesion assay

Rat epithelial cells (IEC-6 line) were grown in 24-well plates in DMEM to confluence; the monolayers were washed twice with PBS, and 250 µl of labeled bacterial cell suspension (at an absorbance of 0.5 (10^6^ CFU/ml) at 600 nm) was added to each well.

Bacterial staining was performed with 10 mM 5-CFDA (5-carboxyfluorescein diacetate) (Sigma, St. Louis, MO) as described by Izquierdo et al. [29]. Briefly, labeled bacterial suspensions were added to IEC-6 cultures at A_600_ 0.50. The epithelial cells and labeled bacteria were incubated together at 37°C for 1 h. IEC-6 cells were washed 2 times with PBS to remove nonadherent bacteria, and adherent cells were lysed in 200 µL 1% SDS (Sigma, St. Louis, MO) in 0.1 M NaOH at 37°C for 1 h [29].

Supernatants were collected in Costar black round-bottom 96-well plates (Corning Inc., Corning, NY, USA), and the fluorescence was measured on a microplate fluorometer (Fluoroskan Ascent, Labsystem, Oy, Finland) with excitation and emission wavelengths of 485 nm and 538 nm, respectively. Adhesion was expressed as the percentage of fluorescence that was recovered from adherent bacteria, relative to the initial fluorescence of the bacterial suspension per well.

### Experimental animals

Wistar-AVN germ-free (GF) rats were reared in Trexler-type plastic isolators under controlled sterile conditions. Granulated gluten-free diet 02 (maize 57%, soya meal 30%, sunflower oil 1.5%, linseed oil 1.5%, salt, DL-lysine 0.5%, DL-methionine 1%, mineral, and vitamin mixture) was sterilized regularly by irradiation (59 kGy, Bioster, Czech Republic) [31], [32].

### Rat intestinal loops

The bacterial strains and gluten were tested in ligated ileal loops of GF rats. Two-month-old GF inbred AVN rats (approximately 200 grams) were deprived of food for the 24 h before surgery (with free access to water). The rats were premedicated intramuscularly with 1 ml of a mixture of ketamine (10 mg/ml) and xylazine (2 mg/ml).

The three ligated loops (each approximately 2 cm long) were created with nylon ligatures in the jejunum and proximal ileum, beginning approximately 3 cm from the ileocecal junction. Each loop was followed by a short intervening segment (2 cm) that was not inoculated [32]. Five hundred microliters of inoculum, containing 10^6^ CFU of bacteria alone or with gliadin (250 µg) and/or IFN-γ (250 U, AbD Serotec), was injected into the intestinal loops. After inoculation, the jejunum was returned to the abdomen, and the laparotomy incision was closed. After 8–9 h, the rats were euthanized by severing of the carotid artery. Tissue samples and contents of the loops were collected for further analysis.

### Immunohistology

Tissue from the loop was fixed immediately in 10% neutral buffered formalin or Carnoy's solution. The fixed tissues were cut and processed using routine methods. Paraffin sections (5 µm) were deparaffinized in xylene, rehydrated through an ethanol gradient to water, and stained in periodic acid-Schiff (PAS) to evaluate mucin-secreting goblet cells. The villi (10–15) in these sections were examined by light microscopy to determine the number of PAS-positive goblet cells per 100 enterocytes in the intestinal tissue, expressed as the medians and quartiles from 5–10 independent measurements.

Gliadin was detected in the intestinal loops by immunolocalization. Briefly, snap-frozen intestinal loop samples, embedded in OCT (Tissue-tek, Sakura Fine Tek, Torrance, CA, USA), were cryosectioned at 6 µm, air-dried, fixed for 5 min in acetone, and stored at −20°C. The sections were washed and endogenous peroxidase blocked by 1% H_2_O_2_. Then, the sections were incubated with peroxidase-labeled monoclonal anti-gliadin antibodies (Elisa Development Prague, Czech Republic) overnight at 4°C, washed, and incubated with Tyramide Signal Amplification - TSA™ Plus Fluorescence system (PerkinElmer, USA) for 30 min. The samples were counterstained with Evans blue and Hoechst to visualise tissue cells and nuclei. Afterwards the sections were embedded in Vectashield mounting medium (Vector Laboratories, UK). All speciemens were examined using a confocal microscope Olympus FV 1000 SIM.

ZO-1 expression was measured by incubating the sections with rabbit polyclonal anti-ZO-1 or anti-claudin-1 antibodies (Zymed Laboratories Inc., San Francisco, CA) at 4°C overnight, washing them, and incubating them with goat anti-rabbit IgG-FITC (in 10% PBS- normal goat serum, Zymed Laboratories Inc.) for 2 h.

Control sections were treated similarly, except that they were incubated with secondary antibodies only. Images of the specimens were viewed under an Olympus BX 40 microscope that was equipped with an Olympus DP 70 digital camera.

### Western blot of tissue lysates

Intestinal tissue from the loops was homogenized on ice in protein extract buffer (Pierce, Rockford, IL) with a protease inhibitor cocktail (Pierce) for 10 min and sonicated. Samples were centrifuged at 10,000× rpm for 10 min at 4°C and stored at −80°C until use. Protein concentrations were measured using the BCA Protein Assay Kit (Pierce).

Proteins were denatured with sample buffer (106 mmol/L Tris-HCl, 141 mmol/L Tris base pH 8.5, 0.51 mmol/L EDTA, 10% glycerol, 2% SDS, 0.22 mmol/L SERVA blue G250, 0.175 mmol/L phenol red, 0.1 mmol/L 2-mercaptoethanol) for 5 min at 100°C, separated on a 10% (for claudin-1) or gradient 5% to 20% (for ZO-1) polyacrylamide gel and blotted onto 0.2-mm PVDF membranes (Serva, Germany).

The membranes were blocked with 2% (w/v) dry milk in 0.05% PBS-Tween-20 for 1 h at room temperature and incubated overnight at 4°C with antibodies against claudin-1 (1∶1000), ZO-1 (1∶1000) (ZYMED Laboratories Inc.), and β-actin (1∶5000) (Abcam, Cambridge, MA, USA). After incubation with the respective primary antibodies, the membranes were washed 3 times for 5 min in 0.05% PBS-Tween-20 and exposed to species-specific horseradish peroxidase-labeled secondary antibodies (1∶1000) (ZYMED Laboratories) for 1 h at room temperature. The reactions were developed using the ECL Plus Western blotting reagent (Pierce), and the signal intensities were measured on the LAS-1000 luminescence detector (Fujifilm, Tokyo, Japan) and processed with AIDA 1000/1D Image Analyzer software, version 3,28 (Raytest Isotopenmmessgeraete GmbH, Straubenhardt, Germany). After stripping, all membranes were re-probed with antibodies against β-actin (1: 5000, Abcam), to document the same protein concentration in all samples.

### Scanning electron microscopy

Terminal ileum tissue was fixed in 3% glutaraldehyde in 0.1 M cacodylate buffer (pH 7.2), dehydrated in an ethanol series (50, 70, 80, 90, 96, 100, and 100% v/v), transferred to 100% (v/v) acetone, and dried in a BALZERS CPD 010 critical-point dryer (Balzers, Lichtenstein). The samples were then sputter-coated with gold and examined on an Aquasem electron microscope (Tescan, Czech Republic) in the SEM mode.

### Rat cytokine array

The cytokine spectra in the rat intestinal loop washes were measured using the semiquantitative RayBio™ Rat Cytokine Antibody Array 1 (RayBiotech, Norcross, GA, USA), which detects 19 growth factors, cytokines, and chemokines, following the manufacturer's recommendations. The signal intensity was measured on an LAS-1000 luminescence detector (Fujifilm), and the resulting images were analyzed using AIDA software (version 3.28; Raytest) to quantify spot densities. The background staining was subtracted, and the data were normalized as described [33].

### Statistical analysis

Statistical analysis was performed using SPSS, version 17.0 (SPSS Inc., Chicago, IL, USA). To establish the homogeneity of variances and the distribution of the data, the Levene test was run. As a result of the non-normal distribution of the data and the nonhomogeneity of the variances, Mann-Whitney *U*-test was used to assess the effect of each variable. The data were expressed as medians and quartiles. Different letters (a–e) mean statistically significant differences between stimuli, the identical letters correspond to non-significant differences. P<0.05 was considered statistically significant.

## Results

### 1. Goblet cell population in the jejunum is influenced by gliadin and intestinal bacteria

**Figure 1:**
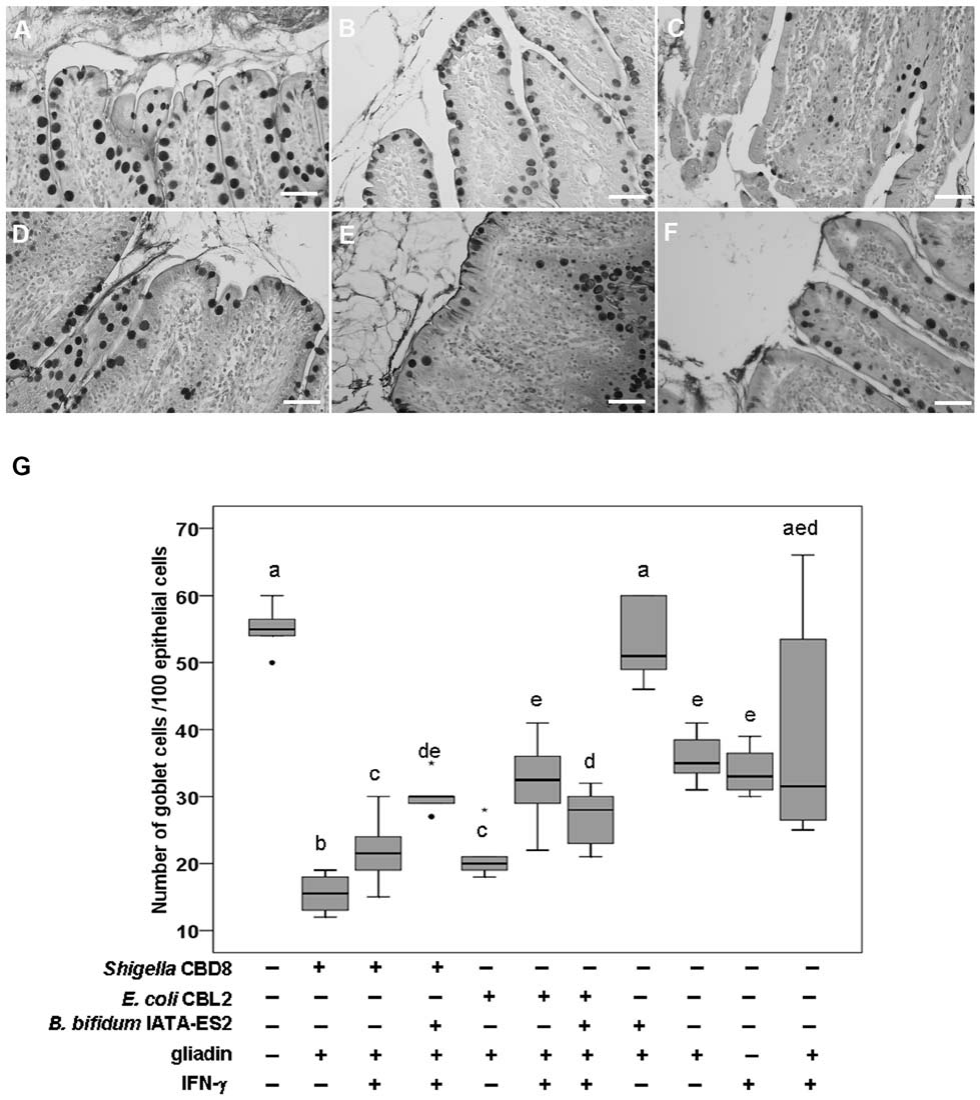
Effects of bacterial strains and gliadin on goblet cells. Histological staining of PAS-positive goblet cells in rat intestinal loops exposed to: *B. bifidum* IATA-ES2+gliadin (200 µg) (A), IFN-γ (225 U) (B), *Shigella* CBD8+gliadin (C), *Shigella* CBD8+gliadin +IFN-γ (D), *E. coli* CBL2+gliadin (E) and *E. coli* CBL2+gliadin+IFN-γ (F). Bacteria were applied at 10^6^/loop. Changes in goblet cells are expressed as medians and interquartile ranges (25% to 75%) of the number of PAS-positive goblet cells/100 epithelial cells (G). These values were for *B. bifidum* IATA-ES2 (39, 35–41), *E. coli* CBL2 (38, 35–41) and *Shigella* CBD8 (25, 20–27) when applied alone to the loops. Different letters (a–e) indicate statistically significant differences between medians as calculated by Mann-Whitney U test (P<0.05). Identical letters correspond to non-significant differences. The separate dots or asterisks indicate outliers. The pictures were obtained for the specimens viewed under an Olympus BX 40. Scale bar, 50 µm.

The effect of gliadin and the proinflammatory cytokine IFN-γ on epithelial cells in the presence or absence of various bacterial strains was examined *in vivo* using loops of small intestine that were ligated surgically from rats kept on a gluten-free diet and reared under germ-free (GF) conditions. As shown in Figure 1, the various stimuli led to changes in the number of PAS-positive goblet cells (examples A–F). To evaluate these changes, the number of PAS-positive goblet cells per 100 epithelial cells was counted (as summarized in Figure 1G). The addition of gliadin into the loops decreased the number of PAS-positive goblet cells compared with PBS used as a control. A similar effect was observed after applying IFN-γ alone and with gliadin (Figure 1B,G). The number of goblet cells after combination of gliadin with *E. coli* CBL2 (Figure 1E,G) or *Shigella* CBD8 (Figure 1C,G) was even lower. The addition of IFN-γ to above mentioned samples slightly increased the number of goblet cells (Figure 1D,F,G).

When gliadin was combined with *B. bifidum* IATA-ES2 (Figure 1A), the number of PAS-positive goblet cells increased, attaining the same value as in PBS-treated loops (Figure 1G). Moreover, the combination of *B. bifidum* IATA-ES2 with *Shigella* CBD8, gliadin, and IFN-γ increased the PAS-positive goblet cell population. The effect of *B. bifidum* IATA-ES2 was less evident when loops were exposed to *E. coli* CBL2 (Figure 1G).

**Figure 2:**
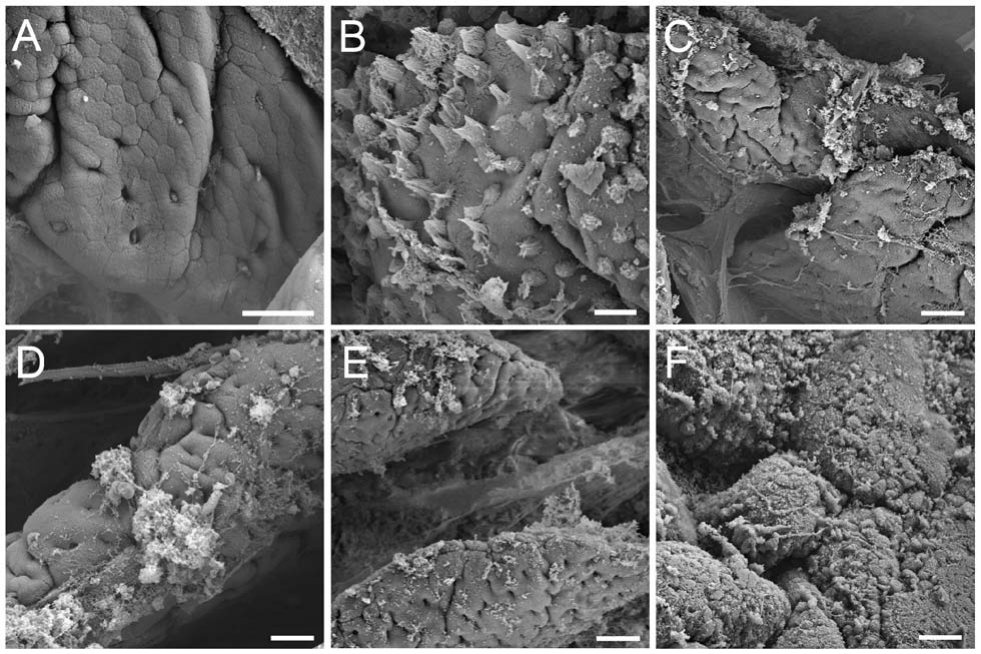
Mucin production by goblet cells in rat intestinal loops. Mucin production after application of *B. bifidum* IATA-ES2 (A), IFN-γ (B), gliadin+IFN-γ+ *B. bifidum* IATA-ES2 (C), *E. coli* CBL2+gliadin+IFN-γ D), *E. coli* BL2+gliadin+ IFN-γ+*B. bifidum* IATA-ES2 (E) and *Shigella* CBD8+gliadin+IFN-γ (F). Bacteria were applied at 10^6^/loop. The samples were coated with gold and examined by Aquasem electron microscopy (Tescan, Czech Republic) in the SEM mode. Scale bar, 20 µm.

By scanning electron microscopy, the addition of *B. bifidum* IATA-ES2 did not affect mucin secretion and did not evoke any changes in intestinal loop architecture (Figure 2A). Mucin secretion was slightly higher after the addition of gliadin (data not shown). IFN-γ, however, induced mucin release (Figure 2B), and higher effect was observed when gliadin and *E. coli* CBL2 were injected with IFN-γ (Figure 2D). The combination of *Shigella* CBD8, gliadin, and IFN-γ boosted mucin secretion into the lumen and impacted the architecture of the epithelial layer, as shown in Figure 2F. Interestingly, addition of *B. bifidum* IATA-ES2 to ”harmful agents” (gliadin and IFN-γ with/without *E. coli)* slightly decreased the mucin secretion as compared to those agents alone (Figure 2C,E).

### 2. Translocation of gliadin into intestinal villi is influenced by intestinal bacteria

We determined whether intestinal epithelial layer permeability and gliadin peptide translocation was affected by bacterial strains. Using mouse anti-gliadin antibody, we monitored the transfer of gliadin peptides through the epithelial layer after exposure of the intestinal loops to IFN-γ and bacterial strains.

**Figure 3:**
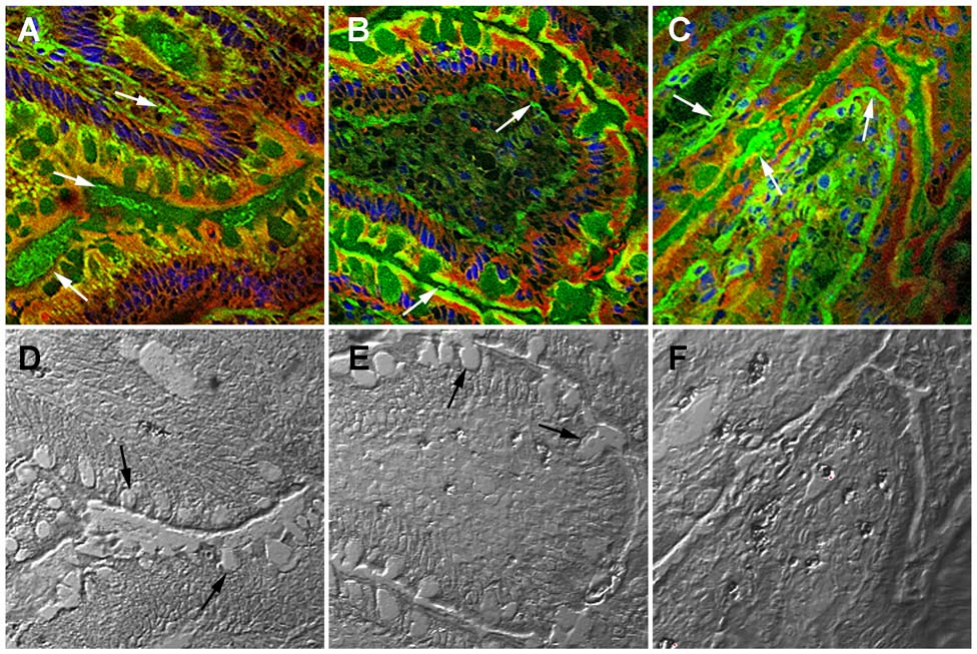
Changes in intestinal permeability induced by gliadin and various bacterial strains. Intestinal loops were exposed to: gliadin fragments with IFN-γ and *B. bifidum* IATA-ES2 (A,D), gliadin+IFN-γ+*E. coli* CBL2 (B,E) and *Shigella* CBD8+gliadin+IFN-γ (C,F); The white arrows indicate gliadin fragments found by immunofluorescence (A–C) using mouse peroxidase-labeled monoclonal anti-gliadin antibody and TSA™ Plus Fluorescence systems and black arrows indicate goblet cells (D, E). Bacteria were applied at 10^6^/loop. The specimens were viewed under confocal microscope Olympus FV 1000 SIM using differential interference contrast (D–F). Scale bar, 20 µm.

Gliadin, when applied with *B. bifidum* IATA-ES2 and IFN-γ, was observed only in low amounts inside the lamina propria - forming foci mainly on the apical section of certain villi (Figure 3A). In contrast, the combination of *E. coli* CBL2, gliadin and IFN-γ induced small, local changes (crypt widening), and gliadin was detected primarily below the epithelial layer (Figure 3B). The combination of *Shigella* CBD8, gliadin, and IFN-γ increased gliadin translocation, and gliadin was detected primarily inside the lamina propria (Figure 3C). Furthermore, using differential interference contrast (Figure 3D–F corresponding to samples in upper row), we confirmed the decrease in a number of goblet cells in loops treated with *E. coli* CBL2 and their loss after treatment with *Shigella* CBD8, documented in Figure 1.

**Figure 4:**
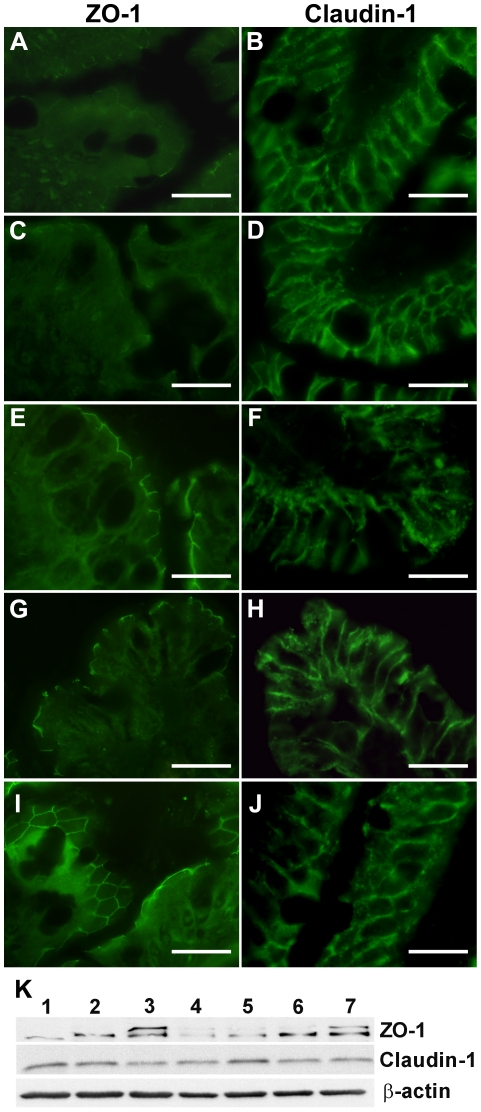
Distribution of claudin-1 and ZO-1 in rat intestinal loops. Exposure of intestinal tissue to gliadin digest alone (A) or with IFN-γ (C) led to reduced ZO-1 expression at the periphery of the villi. The combination of gliadin+IFN-γ+*B. bifidum* IATA-ES2 (E) maintained the original level of ZO-1 as in PBS-treated loops (I). When gliadin+IFN-γ were applied with *E. coli* CBL2 (G) ZO-1 fluorescence was weaker. No changes in claudin-1 localization (B, D, F, H) were detected after any stimulus in comparison with PBS control (J). Representative pictures of three experiments are shown. The specimens were viewed under an Olympus BX 40 microscope. Scale bar, 20 µm. Western blot of tissue lysates (K) from intestinal loops stimulated with: 1. gliadin, 2. gliadin+IFN-γ, 3. *B. bifidum* IATA-ES2+gliadin, 4. *E. coli* CBL2+gliadin +IFN-γ, 5. *E. coli* CBL2+*B. bifidum* IATA-ES2+gliadin+IFN-γ, 6. *B. bifidum* IATA-ES2+gliadin+IFN-γ, and 7. PBS. The separated proteins on membranes were stained with anti ZO-1 or claudin-1 antibodies and re-probed with antibodies against β-actin to document the same protein concentration in all samples.

These data are also consistent with our fluorescence microscopy results, which demonstrated the distribution of TJ components, claudin-1, and ZO-1 in intestinal loops that were treated with gliadin, IFN-γ, and/or various bacterial strains (Figure 4A–J). Gliadin alone or with IFN-γ downregulated ZO-1 expression (Figure 4A,C) compared to PBS-exposed loops (Figure 4I). On the other hand, simultaneous addition of *B. bifidum* IATA-ES2 with IFN-γ and gliadin upregulated ZO-1 expression (Figure 4E). When the loops were simultaneously exposed to *E. coli* CBL2, gliadin and IFN-γ, ZO-1 fluorescence was reduced (Figure 4G).

In contrast, the typical pattern of claudin-1 expression at the periphery of intercellular (enterocyte) contacts was unaffected by addition of gliadin alone or with IFN-γ, *B. bifidum* IATA-ES2 or *E. coli* CBL2 (Figure 4B,D,F,H) compared to PBS-treated loops (Figure 4J). Nevertheless, the combination of gliadin, IFN-γ, and *Shigella* CBD8 nearly extinguished ZO-1 and claudin-1 signals (data not shown).

To support the fluorescence microscopy findings, intestinal tissue from the stimulated loops was extracted, and changes in TJ proteins were measured by western blot. As shown in Figure 4K, ZO-1 expression was more sensitive to various stimuli than claudin-1. Gliadin, IFN-γ, and, particularly, their combination with *E. coli* CBL2 reduced ZO-1 levels in tissues. The addition of *B. bifidum* IATA-ES2 to this mixture increased ZO-1 levels, confirming the fluorescence microscopy data. When *B. bifidum* IATA-ES2 was added with gliadin, ZO-1 levels approximated to those of the PBS control. When *Shigella* CBD8 was used, the fragmentation of TJ proteins was detected (data not shown).

### 3. Interaction of bacteria with the epithelial layer *in vitro*

The different effects of bacterial strains on gliadin translocation and expression might be a consequence of differences in the adhesion properties of individual bacterial strains that determine host-microbe interactions.

**Figure 5:**
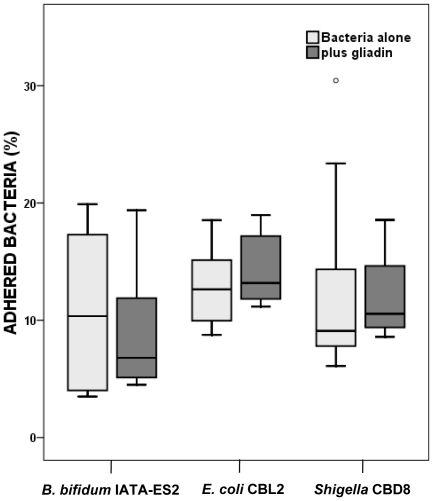
Adhesion of different bacterial strains to IEC-6 cells. The highest percentage of adhered bacteria was observed for *E. coli* CBL2 and *Shigella* CBD8. The differences between tested bacterial strains, as well as the effect of simultaneously added gliadin fragments were non-significant as established by applying the Mann-Whitney U-test. Data are expressed as medians and interquartile ranges (25% to 75%) of adhesion of four independent experiments. None of the differences was found to be statistically significant (P<0.05). The separate dot indicates an outlier.

The interaction of various bacterial strains from celiac patients or healthy subjects (which comprise potentially beneficial and pathogenic bacteria) with epithelial cells was analyzed *in vitro* using the IEC-6 rat cell line; the adherence of bacteria to IEC-6 cells and the impact of gliadin were measured. As shown in Figure 5, the percentage of adhered bacteria varied only slightly, and the differences between *E. coli* CBL2, *Shigella* CBD8 and *B. bifidum* IATA-ES2, were not statistically significant. The simultaneous addition of gliadin fragments and bacteria to cell cultures had an insignificant effect on bacterial adhesion.

### 4. Cytokine secretion into the gut lumen

**Figure 6:**
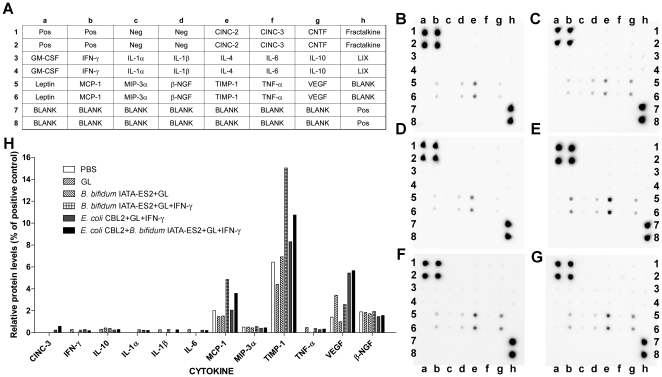
Cytokine array analysis of rat intestinal loops washes. Layout of the arrays (A), cytokine profiles from loops treated with PBS (control) (B), gliadin (C), gliadin+IFN-γ (D), *B. bifidum* IATA-ES2+gliadin+IFN-γ (E), *E. coli* CBL2+gliadin+IFN-γ (F), and *E. coli* CBL2+*B. bifidum* IATA-ES2+gliadin+IFN-γ (G). The data are expressed as relative levels of selected cytokines (percentage of positive controls). Cytokine-induced neutrophil chemoattractant (CINC)-2 and -3, monocyte chemoattractant protein (MCP)-1, macrophage inflammatory protein (MIP)-3α, nerve growth factor β-(NGF), tumor necrosis factor (TNF)-α, vascular endothelial growth factor (VEGF). The signal intensity was measured using the LAS-1000 luminescence detector (Fujifilm, Tokyo, Japan).

Cytokine production in response to administration of food and bacterial antigens and IFN-γ to rat intestinal loops was measured in intestinal washes by cytokine array (Figure 6A–H). The secretion of cytokines, such as chemotactic factor for monocytes and neutrophils (MCP)-1, tissue inhibitor of metalloproteinase (TIMP)-1, vascular endothelial growth factor (VEGF), and beta-nerve growth factor β-(NGF), increased.

The most abundant cytokines, MCP-1 and TIMP-1, which play a role in tissue protection, were induced by *B. bifidum* IATA-ES2 in a mixture of gliadin and IFN-γ. The addition of *E. coli* CBL2 to this mixture decreased MCP-1 and TIMP-1 release into the intestinal loops. VEGF secretion rose, particularly by the addition of *E. coli* CBL2 to gliadin and IFN-γ but was unaffected by simultaneous addition of *B. bifidum* IATA-ES2 to this mixture.

The spontaneous production of β-NGF was independent of any stimulus. Further, cytokine-induced neutrophil chemoattractant (CINC)-3, IFN-γ, IL-10, IL-1α, IL-1β, IL-6, macrophage inflammatory protein (MIP)-3α, and TNF-α levels were low. Although it was difficult to determine the effect of the stimuli on low cytokine production, CINC-3 was detected only in loops that were inoculated with *E. coli* CBL2. In PBS treated loops the cytokine IL-10, IL-1α, IL-1β, and TNF-α were undetectable (as summarized in Figure 6H).

When *Shigella* CBD8 replaced *E. coli* CBL2, cytokine levels increased markedly. Nevertheless, the high background of the microarrays, reflecting the impact of *Shigella* CBD8 on intestinal tissue, rendered the precise evaluation of these data impossible.

## Discussion

There is limited data on the effects of bacteria and their components on the intestinal barrier and the immune response to dietary proteins. In this study, we observed the effects of potentially pathogenic bacterial strains, isolated from the feces of celiac patients or bifidobacteria, on gliadin- and IFN-γ-induced immune reactions.

Gliadin, when applied into the intestinal loops of germ-free rats with the Gram-negative bacterial strain *E. coli* CBL2 and *Shigella* CBD8, significantly reduced the number of PAS-positive goblet cells in the jejunum; the opposite effect was observed when *B. bifidum* IATA-ES2 was applied. The decreased caused by gliadin alone was nearly completely reversed by the addition of *B. bifidum* IATA-ES2. Moreover, the decline of PAS-positive goblet cell population that was caused by gliadin and *E. coli* CBL2 or *Shigella* CBD8 was lower when they were combined with *B. bifidum* IATA-ES2 and/or IFN-γ. The decrease in number of goblet cells appeared to be caused by massive mucin secretion or cell exhaustion, accompanied by changes in jejunal architecture, similar to the changes that occur in the early stages of CD [34].

The direct effect of intestinal microbiota on the number of PAS-positive goblet cells and on the composition and secretion of mucins occurs on colonization of GF animals - namely, mice and rats. In GF rodents, goblet cells were shown to be fewer in number and smaller in size and mucus layer is thicker compared with conventionally raised animals. In rats that are raised under GF conditions and inoculated with human fecal microbes (human microbiota-associated rats), the number of mucin-containing goblet cells in the small intestine is higher than in conventionally raised rats [35]–[37].

Commensal and pathogenic bacteria and bacterial LPS induce host goblet cells to produce glycosylated mucins that are digestible and beneficial for their own metabolism. An example is the monoassociation of GF mice with wild-type *Bacteroides thetaiotaomicron* (gut commensal), which induce the production of fructosylated glycoconjugates, used by the bacterium as a nutrient source [38]–[41].

Studies have shown that dietary factors affect goblet cell numbers and modulate their secretory activity [37], [42], [43]. The activating property of gliadin was also demonstrated *in vivo* in GF rats; where repeated oral administration of gliadin to neonatal rats led to effects like colonization with SPF (specific pathogen-free) microbiota [32]. In earlier reports, increased glycoprotein synthesis in jejunal tissue were observed in untreated celiac patients [44], [45].

Our finding of mucin secretion by goblet cells, as documented by scanning electron microscopy, suggests that IFN-γ induced secretion is partially compensated by increased mucin synthesis. The markedly increased mucin secretion that is induced by enterobacteria with gliadin and IFN-γ, however, is accompanied by a decrease in the number of PAS-positive goblet cells, damage to tight junctions, and remodeling of the epithelial layer.

Recently, the effect of gliadin on the epithelial layer was noted in *in vitro* studies using epithelial cell lines. Exposure to peptic-tryptic fragments of gluten or gliadin leads to increased permeability of Caco-2 monolayers, a human colon epithelial cell line, due to lower expression of TJ proteins [21], [22], [46], [47]. Our experiments with rat intestinal loops confirmed the decreased expression of the TJ protein ZO-1 after *in vivo* stimulation with gliadin, IFN-γ, and/or enterobacteria from CD patients by immunofluorescence and western blot. The second protein band reacting with anti ZO-1 antibodies in some samples, also shown by others [48]–[50], could be a consequence of partial aggregation, complex formation, or external stimuli. In addition, our results demonstrate that these adverse effects are partially restored by *B. bifidum* IATA-ES2.

We noted a spectrum of cytokines in the intestinal washes after various stimuli. Secretion of TIMP-1 (inhibitor of metalloproteinase, an enzyme of the endopeptidase family, important in resorption and remodeling of extracellular matrix) was decreased after gliadin treatment and increased after the addition of *B. bifidum* IATA-ES2 and IFN-γ. The effect of gliadin is consistent with the upregulation of intestinal metalloproteinases and changes in TIMPs in patients with celiac disease and dermatitis herpetiformis [51]–[53].

In a recent study, we observed that the two enterobacteria studied *E. coli* CBL2 and *Shigella* CBD8, induced proinflammatory signals in PBMCs (peripheral blood mononuclear cells) through an intact epithelial barrier (Caco-2 cells). This property appeared to be associated with the pathogenic potential of the strains. Stimulation of Caco-2 cells with other *Bifidobacterium* strains did not exert similar effects, confirming that the intestinal epithelial cells provided a physical barrier, preventing overstimulation and inhibiting monocyte activation [54].

It has been suggested that the beneficial effects of bifidobacteria are related to their ability to adhere to the epithelial layer, preventing the adhesion of pathogenic bacteria. Yet, the potentially pathogenic strains that we tested have similar adhesion properties as *B. bifidum* IATA-ES2. The adhesion of pathogens to host tissues might be a potentially negative hallmark, especially adhesion to the damaged tissue, which is often the first step in pathogenesis [55], [56].

In conclusion, our data in GF rat intestinal loops highlight the potential for gliadin fragments and/or IFN-γ to reduce the number of PAS-positive goblet cells and increase mucin secretion; changes typical for early stages of enteropathies in general. Interestingly, the changes induced by gluten and IFN-γ were more pronounced when these agents were combined with potentially pathogenic enterobacteria. The decrease in PAS-positive goblet cells by gliadin was reversed in the presence of *B. bifidum* IATA-ES2. Moreover, enterobacteria can contribute to the translocation of gliadin fragments into intestinal loops and to changes in ZO-1 expression. Interestingly, *B. bifidum* IATA-ES2 has beneficial effects on cytokine secretion into intestinal loops, upregulating chemotactic factors and inhibitors of metalloproteinases and thus contributing to gut mucosal protection. Therefore, we hypothesize that the composition of the intestinal microbiota and the presence or absence of specific bacteria could play a role in CD pathogenesis.
